# Correction: The role of thermal annealing on the microstructures of (Ti, Fe)-alloyed Si thin-film anodes for high-performance Li-ion batteries

**DOI:** 10.1039/c8ra90038h

**Published:** 2018-05-22

**Authors:** Minsub Oh, Ilhwan Kim, Hoo-Jeong Lee, Seungmin Hyun, Chiwon Kang

**Affiliations:** School of Advanced Materials Science and Engineering, Sungkyunkwan University Suwon Republic of Korea chiwonkang@skku.edu; Nano-Convergence Mechanical Systems Research Division, Korea Institute of Machinery and Materials (KIMM) Daejeon Republic of Korea hyun@kimm.re.kr

## Abstract

Correction for ‘The role of thermal annealing on the microstructures of (Ti, Fe)-alloyed Si thin-film anodes for high-performance Li-ion batteries’ by Minsub Oh *et al.*, *RSC Adv.*, 2018, **8**, 9168–9174.

The authors regret that the name of the co-author Ilhwan Kim was spelled incorrectly in the original article. The correct spelling is presented herein.

The Royal Society of Chemistry apologises for these errors and any consequent inconvenience to authors and readers.

## Supplementary Material

